# Anticipatory Guidance: Developing a Patient Navigation Pathway to Reduce the Financial Toxicity of Cancer

**DOI:** 10.18103/mra.v11i10.4582

**Published:** 2023-10-25

**Authors:** Christabel K. Cheung, Laundette Jones, Haelim Lee, Jordan N. Bridges, Reginald Tucker-Seeley, Melissa Ana Liriano Vyfhuis, Maria C. Gianelle, Bria N. Thomas, Gail Betz, Laurie Waldo, Alan S. Hirsch, Shana O. Ntiri

**Affiliations:** 1 Assistant Professor, University of Maryland School of Social Work; 2 Associate Professor, University of Maryland School of Medicine; 3 Doctoral Research Assistant, University of Maryland School of Social Work; 4 Graduate Research Assistant, University of Maryland School of Social Work; 5 Principal Owner, Health Equity Strategies and Solutions; 6 Adjunct Assistant Professor in Radiation Oncology, Baltimore Washington Medical Center; 7 Doctoral Research Assistant, University of Maryland School of Medicine; 8 Doctoral Research Assistant, Temple University School of Podiatric Medicine; 9 Research and Education Librarian, Health Sciences & Human Services Library, University of Maryland, Baltimore; 10 Social Worker, University of Maryland Medical Center; 11 Oncology Social Work Team Lead, University of Maryland Medical Center; 12 Associate Professor, Department of Family and Community Medicine, University of Maryland School of Medicine

## Abstract

**Background:**

Healthcare providers have an influential role in the experience of financial toxicity among their cancer patients, yet patients commonly report unmet needs and dissatisfaction regarding communication with their providers about financial concerns.

**Aims:**

The purpose of this study is to develop a novel financial navigation pathway that leverages existing patient financial services and resources with corresponding patient-centered, community-informed strategies, via study participants, that may be utilized in routine care to reduce financial hardship among cancer patients.

**Methods:**

We conducted in-depth interviews (n=50) with 34 cancer patients and 16 cancer care professionals at a National Cancer Institute designated comprehensive cancer center located in a dense urban area of the US between December2022 to June 2023.

**Results:**

Content analyses resulted in emergent themes and representative quotations on experiences of financial hardship within the material, behavioral, and psychosocial domains. Investigators used emergent themes to develop financial strategies and construct a financial navigation pathway to screen patients for and intervene upon the financial toxicity of cancer in routine care.

**Conclusion:**

This study followed an innovative approach by constructing a financial navigation pathway tool that follows the oncological workflow at a National Cancer Institute designated comprehensive cancer center. Future research is needed to test the tool’s impact on financial toxicity, cancer outcomes, and other health-related outcomes, and to better understand how much patient navigation is needed to bring about meaningful change.

## Introduction

In the United States, the financial hardship of cancer and its treatments is prevalent and has a profound impact on quality of life for patients and their family members.^1^

Over the past decade, *financial toxicity*, defined as a patient’s problems related to medical costs that may lead to debt and bankruptcy, diminished quality of life, and difficulties accessing medical care,^2^ has become accepted as a clinically relevant patient-reported outcome in oncology.^3^ The literature largely shows an association of cancer-related financial hardship with increased mortality risk,^4,5^ elevated biological markers of inflammation,^6^ health disparities and poor outcomes,^7,8^ poor mental health outcomes,^9,10^ and reduced utilization of available health care.^11^ Healthcare providers have an influential role in the experience of financial hardship among their cancer patients. Hong et al’s study of the 2016–2017 Medical Expenditure Panel Survey’s nationally representative data^12^ found that cancer patients who reported having a detailed discussion with their providers about cancer care costs, on average, spent 33.8% less on out-of-pocket health care expenses across all age groups with patients ages 18–64 years seeing greater savings (than their older counterparts ages 65+).^12^ Even still, cancer patients commonly report unmet needs and dissatisfaction regarding communication with their providers about financial concerns.^13^

Moreover, data from our own cancer center points to racial disparities related to unmet financial concerns. Specifically, Kronfli et al’s study (n=266) of patients undergoing radiation therapy with curative intent at our National Cancer Institute (NCI)^14^ designated comprehensive cancer center showed that significantly higher proportions of Black versus non-Black patients reported unmet needs regarding pain, stress management, and transportation. Multivariable results showed that Black patients were 2.6, 2.2, 7.2, and 3.4 times more likely than non-Black patients to request assistance with pain, stress, transportation, and financial aid, respectively.^14^

### Screening and Assessment of Financial Toxicity

In 2017, Khera et al’s commentary in *Cancer* on the financial burden of cancer treatment^15^ argued for universal financial distress screening in routine cancer care to be as ubiquitous as psychological distress screening. A provocative challenge at the time that continues to hold. Early and effective screening for financial toxicity in cancer care delivery is critical to equipping clinical teams with information necessary to develop patient-centered interventions that potentially improve patients’ experiences.^16,17^

Despite mounting evidence on the prevalence of financial hardship, only a small proportion of comprehensive cancer centers utilize financial screening in routine care. A recent study evaluated the implementation of distress management guidelines by 15 of the 18 National Comprehensive Cancer Network (NCCN) member institutions.^18,19^ Findings revealed that only 20% of comprehensive cancer centers screened all patients for psychological distress (including financial hardship) per National Comprehensive Cancer Network guidelines. Extant studies suggest that, due to time constraints and competing demands, oncology providers may rely on their personal clinical judgment and avoid using distress screening tools.^20–22^

A review of distress screening and management tools in cancer care suggests that initial screening for psychosocial distress should be introduced at the point of entry into cancer care, thus facilitating proper referral to psychosocial oncology specialists for more detailed assessment.^23^ Additionally, as patients move along the oncological treatment pathway, serial monitoring of evolving distress associated with practical problems such as financial hardship is necessary because the economic burden of treatments builds up from accumulating treatment costs and likely disruption to occupational engagement related to earned income.^23^

According to a recent study of social workers and financial care counselors at a cancer center, barriers to effective utilization of financial resources include frustration over the lack of financial resources, increasingly stringent eligibility criteria, process inefficiencies, limited resources to identify at-risk patients, and inadequate insurance coverage and availability.^24,25^ It follows that the development of effective and durable strategies to address financial hardship must be informed by patient and community engagement to address these issues. Furthermore, developers must respond to evidence showing that cancer-related financial problems are disproportionately represented by patients who are younger, members of a racial minority group, and have a higher treatment burden, and that these financial problems are found to contribute to cancer patients forgoing or delaying necessary medical care.^26–29^

### Conceptual Frameworks

**Figure 1.**
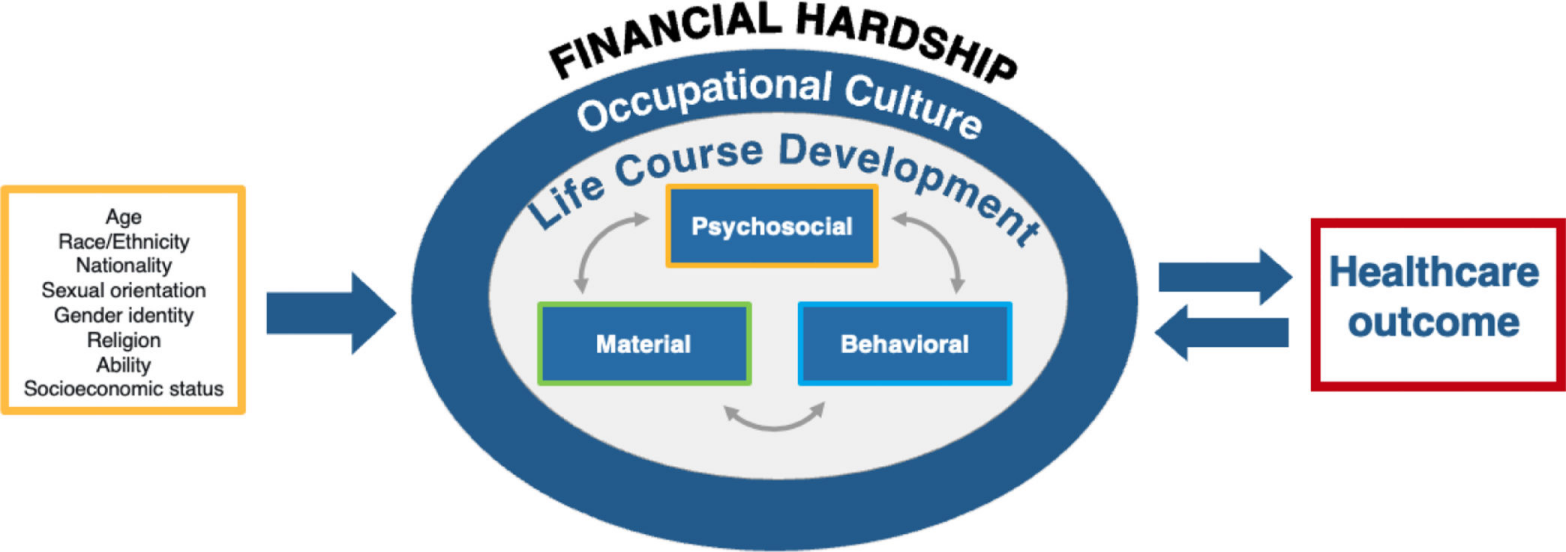
Tucker-seeley’s conceptual framework of the financial hardship of cancer^1^ ^1^ From Cheung CK, Nishimoto PW, Katerere-Virima T, Helbling LE, Thomas BN, Tucker-Seeley R. Capturing the financial hardship of cancer in military adolescent and young adult patients: A conceptual framework. *J Psychosoc Oncol.* 2022;40(4):473–490. doi:10.1080/07347332.2021.1937771

The current study was informed by two conceptual frameworks and one set of guiding principles. First, we used Tucker-Seeley’s expanded conceptual framework of the financial hardship of cancer^30,31^ as the analytical framework to inform our participant interview guides and to examine the relationships between reported experiences (Figure 1). Based on theories developed to explain health disparities, Tucker-Seeley’s framework of financial hardship after a cancer diagnosis describes experiences occurring within three domains—material, psychosocial, and behavioral—that are situated within the contexts of life course development and occupational culture. Altogether, these aspects may have a bidirectional relationship with a patient’s health outcomes.^30,31^ This framing also builds upon evidence showing that the measurement of socioeconomic status requires multi-dimensional assessments. Furthermore, results from Surachman et al’s exploratory factor analysis of financial hardship support Tucker-Seeley’s three-domain model.^6^

Second, we used Dr. Harold P. Freeman’s Harlem Model’s^32^ nine principles for excellence in patient navigation to guide the development of a financial navigation pathway and compose financial navigation strategies that respond to real-world experiences. The central mission of Freeman’s principles is “to promote the timely movement of an individual patient through an often-complex health care continuum,” with their core function being “the elimination of barriers to timely care across all phases of the healthcare continuum.”^32^

Third and finally, we used the Donabedian Model^33–35^ of care to guide the description of key aspects of a financial navigation pathway. The Donabedian quality of care framework is the dominant paradigm, among existing health care coordination frameworks,^36^ for assessing the quality of health care. The framework collects information on three components that are related to each other linearly from left to right: the structural context in which care is delivered; the process by which providers provide care to patients; and the resulting health outcomes pathway. Capturing these three domains of the Donabedian Structure-Process-Outcome Model^37^ ensured that our resulting financial navigation pathway could later be tested for effectiveness and quality improvement against this framework in future research.

### Study Purpose

The current research was initiated based on a preliminary interview with the social work team at a National Cancer Institute (NCI) designated comprehensive cancer center located in the dense urban city of Baltimore (University of Maryland Greenebaum Comprehensive Cancer Center). The interview revealed that, between January 1 to April 1, 2020, the social work team received 96 referrals for financial distress from health care providers at the cancer center. Of the 96 patients, only 21 were referred to patient navigation services for help with various treatment-related financial assistance needs. Additionally, the social work team receives an average of 52 patient referrals per month for transportation assistance but does not track whether or how social workers follow-up with these patients. These data suggest that there is some level of screening and referral happening, but no system in place to ensure equitable access among patients. There are currently no clinical processes nor guidelines at the cancer center for systematically identifying patients in need of financial navigation, prioritizing referrals, and offering financial strategies.

The purpose of this study is to develop a novel financial navigation pathway that will leverage existing patient financial services and resources with corresponding patient-centered, community-informed strategies, via study participants, that may be utilized in routine care across the cancer care continuum to reduce financial hardship among patients at a National Cancer Institute designated comprehensive cancer center. Our primary research question asks: How can the oncological patient treatment pathway be utilized by the interprofessional care team to reduce cancer-related financial hardship in its material, psychosocial, and behavioral domains? Guidance from this tool centers on utilization of the oncological patient treatment pathway by the interprofessional oncology care team to intervene upon the financial toxicity of cancer.^30,31^

Throughout this manuscript, the terms financial hardship, financial distress, and financial burden are used interchangeably in reference to challenging experiences with severity that may or may not build to a toxic level-i.e., *financial toxicity.*

## Methods

The current exploratory phenomenological study^38^ applied the Framework Method for qualitative content analysis in multi-disciplinary health research.^39^ The combined effects of phenomenological epistemology and the Framework Method enabled our investigator team to use a conceptual framework of financial hardship^31^ and guiding principles of patient navigation^32^ to construct an analytical framework to organize participant interviews and qualitative analysis. Within the domains of the analytical framework, textual data from interviews were sorted, coded, and abstracted to produce emergent themes that capture and illustrate the essence of participants’ complex experiences of financial hardship across the cancer care continuum.

### Participants and Recruitment

Fifty participants (n=50), comprising 34 patients and 16 cancer care professionals (Table 1 ), were recruited via purposive sampling and snowball techniques using electronic flyers that were distributed across the professional networks of members of our investigator team. Eligible patient participants were cancer patients over the age of 18 years, who were treated within the cancer center’s catchment area and willing to share their personal experiences of cancer-related financial hardship. Eligible cancer care professional participants were individuals employed within the cancer center’s catchment area with responsibility for the clinical care of cancer patients. As a token of appreciation, each study participant received a $50 e-gift card upon completion of their interview.

### Data Collection

Each participant interview took between 30 to 90 minutes to complete, and interviews were conducted from December 2022 to June 2023. Five members of the research team (C.K.C., H.L., B.N.T., M.C.G., & J.N.B.) participated in conducting interviews. This study was approved by the institutional review board priorto data collection. Interviews were audio-recorded with the participant’s approval and transcribed by Otter.ai software. Electronically created transcripts were then verified for verbatim accuracy by four members of the investigator team (H.L., B. N.T., M.C.G., & J.N.B.).

Table 2 displays the two interview guides used to pose open-ended questions to cancer patient participants and cancer care professional participants, respectively. The focus areas within each interview guide were informed by Tueker-See ley’s conceptualization of financial hardship,^30,31^ Freeman’s patient navigation principles,^32^ and Donabedian’s theorizations of structure-process-outcomes for health care quality.^37^ Interview questions were generated after a comprehensive literature search and discussions among members of the investigator team to arrive at appropriate wording for comprehensibility to our study participants.

### Data Analysis

Transcripts were analyzed by five members of the research team (C.K.C., H.L., B.N.T., M.C.G., & J.N.B.). The first stage of content analysis involved verification of transcripts for accuracy. Next, investigators listed and identified significant statements made by study participants. All significant responses that reflected financial hardships along the cancer care continuum were extracted to form codes that were subsequently clustered and abstracted into themes. Codes and themes were compared within and across interviews to investigate the nature of financial hardship experienced by cancer patients. For each domain of financial hardship, representative quotations of the most salient themes were extracted and grouped (Table 3).

Common themes that emerged from coding participant responses are presented in Tables 4, 5, and 6. Tables 4 and 5 present emergent themes organized within Tucker-Seeley’s material, behavioral, and psychosocial domains of financial hardship.^30,31^ Emergent themes and subthemes related to patient navigation,^32^ and health care quality^37^ are shown in Table 6.

Three qualified members of the research team employed by the cancer center in clinical care and principal investigator roles (L.J., S.O.N., & M.A.L.V) confirmed relevant financial strategies as well as the timing of these strategies for each emergent theme. After quotes and themes related to financial hardship experiences were extracted, investigators utilized interview data to develop strategies for mitigating financial toxicity of each emergent theme and construct a final resulting financial navigation pathway.

## Results

### Participant Characteristics

Table 1 displays the sociodemographic characteristics of our final study sample (n=50). The majority of cancer patient participants (n=34) reported being diagnosed with breast cancer (71%, n=24), and self-identified as Black (62%, n=21), non-Latinx (94%, n=32), and female (85%, n=29). The mean age of participants at the time of their initial cancer diagnosis was 43-years-old, and their mean age at the time of this study was 50-years-old. Nearly half of the patient participants held a graduate degree (47%, n=16) and over one-third had an annual household income of $75,000 or over (35%, n=12).

Among cancer care professionals (n = 16) participating in this study, more than half identified as White (56%, n=9), non-Latinx (69%, n = 11), and female (63%, n=10). The mean age of cancer care professionals at the time of our study was 36-years-old, and on average, these participants had held their current professional roles over the past 4.7 years.

### Material Domain

A total of 10 themes emerged regarding financial challenges in making ends meet (Table 4). One of the primary financial issues reported by patients and cancer care professionals in the material domain was the burden of insufficient, lack of, or loss of health insurance coverage, which they attributed to being a low-wage earner. The second and third most common themes were “Health insurance issues with mounting medical bills and household bills” and “Employment problems including job loss, job lock, and inability to work due to cancer.”

One patient described difficulty meeting material needs while undergoing active treatment. She said, “It was challenging to make ends meet. And that does include not going to doctor’s visits … nobody can live off one bra.” Another patient also expressed how health insurance plays a substantial role in depleting their savings.

Out of like my AmeriCorps insurance, I got it as long as I could. And then for the rest of that year, I paid health insurance out of my pocket. Because it was better insurance than what I could get like going on whatever disability like Medicare. So, I did that. And that completely depleted my savings.

Financial hardship persisted among patients even after completing cancer treatments. A patient participant explained, “Once I was post-treatment, my job … they fired me because of my cancer … that’s where the hardship actually kind of began.”

One cancer care professional pointed out that they see a lot of single patients who express concerns about unemployment, extended unpaid leave, and financial instability amplified by a lack of social support. Another cancer care professional highlighted concerns regarding the overall well-being of patients, emphasizing their struggle to make ends meet and the consequential challenge of attending crucial medical appointments.

We see a lot of patients whose parents might either stop working completely or take, you know, an extended unpaid leave from work. And we see a lot of associated hardships with that financially. You know, trouble making ends meet to pay their rent; trouble, you know, paying for utilities.

When patients were asked about material hardship experienced by their family or household members, a total of seven common themes emerged (Table 5). The themes described how family or household members were asked to contribute practical support, felt pressured to help, and felt burdened by requests to make financial contributions while the needs of their financial dependents increased. Participants commonly shared that they had to ask their family members to provide practical support or make financial contributions.

I did live with my parents during treatment because I needed care…they were already paying for my food; they were already paying for like transportation costs. I mean, they didn’t ask me for anything. They paid for gas; they drove; they picked up medication.

One cancer care professional shared experiences of family members showing anger or resentment towards patients due to accumulated caregiving responsibilities. She said, “A lot of resentment between spouses. You know, there’s already like, some problems there with transitioning from being like ‘lovers’ spouses almost into like a parental role in a way, because they’re having to care for their loved one.”

### Behavioral Domain

Across patient participants, seven common themes emerged related to behavioral changes that patients made to adjust to their new financial situation (Table 4). The three most salient themes were “Reduced personal discretionary expenses,” “Took on additional work to create new income,” and “Changed living situation.” One patient said, “I stopped doing a lot of impulse buying, you know, luxury items, clothing … stuff like that… I didn’t really have the energy to go out, like I used to.” Additionally, some patients reported moving back to their family or parents’ house to deal with the new financial difficulties.

Cancer care professionals in our study emphasized concerns about the potential for behavior adjustments to inadvertently interrupt or obstruct patients’ access to essential medical care. They explained that changes may potentially create a detrimental cycle that jeopardizes the patients’ health and overall well-being.

So, I think one of the toughest trade-offs, that I’ve actually heard from my own work, is the fact that some cancer patients may actually decide not to get treated… or not to continue their treatment in order to be able to pay for these needs.

Major behavioral changes among patient’s family or household members were also detailed and resulted in a total of seven common themes that pertained to family/household members shouldering additional financial responsibilities on behalf of cancer patients (Table 5). For instance, one patient shared, “I needed my mom’s support while I was recovering from surgery and stuff. So, there were times that she was home with me without receiving pay … picking up medication and stuff during that time.”

### Psychosocial Domain

A total of seven common themes emerged in the psychosocial domain (Table 4). Patient participants in our study encountered a diverse range of psychological distress and found it difficult to distinguish between the emotional impacts stemming from the physical illness of cancer and those arising from financial concerns. The majority of patients explained that their financial challenges of cancer worsened the already existing stress of navigating their cancer treatments and survival. Some participants shared feeling guilt and shame associated with their cancer diagnosis, and others even admitted to contemplating thoughts of suicide. One patient shared, “It’s a different kind of helplessness. A big thing that … or at least in my experience … that cancer does is it takes away a lot of your control.”

The psychosocial stresses of cancer also affected cancer patients’ family/household members. A total of seven common themes emerged. The most described experiences included emotional conflicts among family and/or household members, family members providing emotional support to the patient, and children being worried about finances.

### Critical Moments of Financial Hardship Following a Cancer Diagnosis

When participants were asked about the most challenging phases of financial hardship across the cancer care continuum, all four stages of detection, diagnosis, treatment, and survivorship were mentioned. However, both patients and cancer care professionals emphasized the significance of financial challenges in off-treatment survivorship due to the persistent financial challenges of cancer that outlast the active treatment period (Table 6).

Among 13 subthemes, notable moments of financial hardship addressed by participants were when they experienced the co-occurrence of the financial hardship of cancer and the strain of being low-income; when side effects surfaced; and when informal support was less present. For patients who were already grappling with precarious financial situations prior to their cancer diagnosis, the burden of cancer worsened preexisting financial hardships, exacerbating the strain on their economic well-being (Table 3). When asked about critical moments of financial hardship fortheir patients, cancer care professionals mainly highlighted the impact of cancer recurrence on patients’ financial well-being stating, “I think that what people don’t kind of realize is that [even though] diagnosis is one stage, what we see at our cancer center is a lot of like relapsed and refractory patients.” Professional participants described observing more intense financial struggles and more greatly depressed emotional well-being among cancer patients with relapsed or refractory disease.

### Helpful Financial Assistance and Resources

When participants were asked about financial assistance they utilized or found helpful in addressing financial challenges, 12 subthemes emerged. The most helpful assistance included direct financial support from family, help accessing financial resources from family or friends, and utilizing a family member’s health insurance benefits.

### Advice from Cancer Patients

The three most common pieces of advice from patient participants to cancer care professionals were to “Increase awareness about patients’ social and financial circumstances,” “Increase knowledge of patient needs before treatment,” and “Provide financial information before treatment begins.” Likewise, patient participants offered advice they wished to convey to fellow cancer patients with an overarching message to actively address their personal needs and not be discouraged nor hesitant to seek help. The three most prominent subthemes on advice to other cancer patients were, “Ask for practical and financial assistance,” “Create robust professional and personal support networks,” and “Contact insurance providers to understand your coverage and available resources.”

## Discussion

Cancer patients are sacrificing their financial health for survival from life-threatening illness and vice versa, while their family and/or household members are often involved in this dilemma. Results from this study reveal common themes of financial hardship experienced by patients and their family/household members in the domains of material, behavioral, and psychosocial issues following a cancer diagnosis. Additionally, participants described critical moments throughout the cancer care continuum when these challenges occurred.

Taken together, findings from content analysis of in-depth interviews in our study demonstrate that an effective *financial navigation pathway* for cancer patients at a National Cancer Institute designated comprehensive cancer center must prioritize early integration of financial toxicity screening, frequent assessment, and intervention strategies that respond directly to patients’ experiences, as well as concerted collaboration along a financial navigation pathway among the interprofessional care team. Figure 3 presents a patient-centered and community-informed financial navigation pathway developed from our findings.

**Figure 2.**
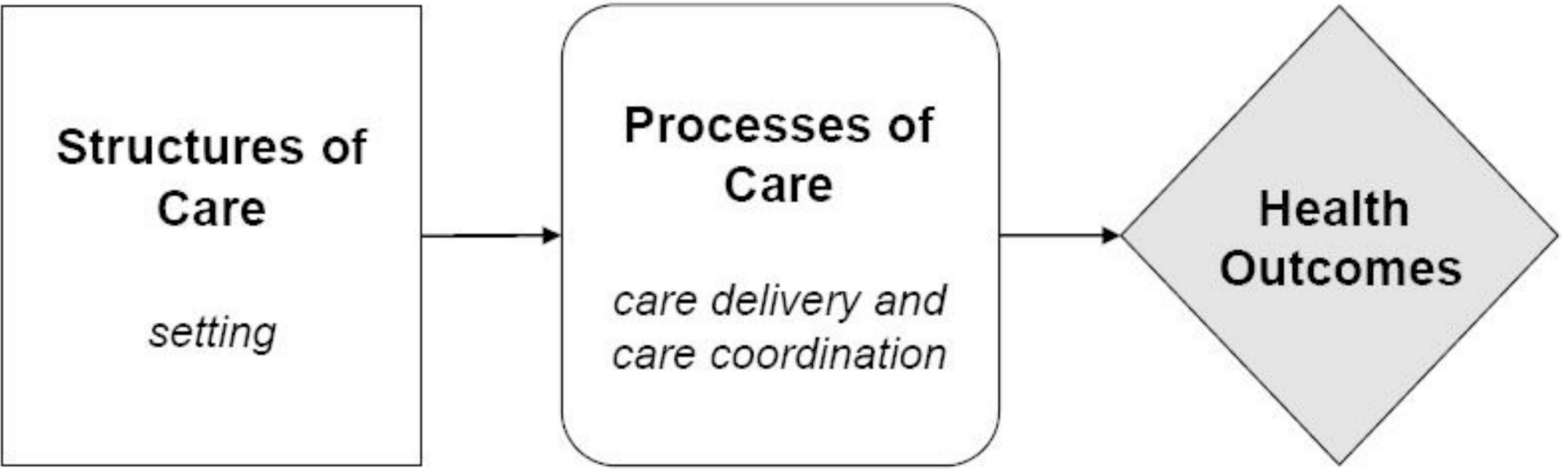
Donabedian’s quality of care framework^1^ From Agency for Healthcare Research and Quality (US); 2007 Jun. (Technical Reviews, No. 9.7.) [Figure], Figure 4. Donabedian’s Quality Framework. https://www.ncbi.nlm.nih.aov/books/NBK44008/fiaure/A25995/

Our investigator team applied the Donabedian Structure-Process-Outcome (SPO) model^37^ (Figure 2) by outlining the workflow structures, processes, and outcomes of a financial navigation pathway aimed at intervening upon the financial toxicity at critical moments across the cancer care continuum. In doing so, the financial navigation pathway tool can be readily examined for ongoing quality improvement and tested for effectiveness in future research.

First, the ‘Structure’ within which the financial navigation pathway is situated is the National Cancer Institute designated University of Maryland Greenbaum Comprehensive Cancer Center located in the dense urban city of Baltimore and the University of Maryland Greenbaum Cancer Care Continuum.^40^ The cancer center’s catchment area of Baltimore City and 10 surrounding counties in the state of Maryland comprises approximately 5.4 million residents disproportionately impacted by cancer, and the region is characterized by concentrated areas of poverty, and gentrification.

Second, the ‘Process’ is provided by recommended financial intervention strategies (e.g., screening, assessment, counseling, and referrals, etc.) for cancer care professionals to employ, which may be monitored for consistency. Third, the ‘Outcomes’ are the themes related to patients and their family/household members’ experiences of financial hardship within the material, behavioral, and psychosocial domains, which may be examined to report the impact of the financial navigation pathway on the financial toxicity of cancer.

### Universal Screening for Financial Distress

Due to the high volume of patients with financial concerns at the cancer center, the National Comprehensive Cancer Network (NCCN) Guidelines Version 2.2023 Distress Thermometer and Problem Checklist (Distress Thermometer) ^41^ will be used to universally screen for financial distress upon initial intake of all cancer patients at the point of entry into the cancer center. The Distress Thermometer screens for financial distress by asking patients to indicate the level of unpleasant experiences they have had in the past week on a scale of 0–10. The tool also contains a problem list that asks patients to indicate any specific concerns by marking all that apply to list of various concerns and provides an open-ended free write option for other concerns.

We recommend that Distress Thermometer financial distress screenings be driven and led by a non-physician member of the oncology care team (e.g., physician’s assistant, nurse practitioner, infusion/radiation nurse, etc.) to be assigned according to the workflow of the clinic. Ideally, an electronic version of the Distress Thermometer would be completed by the patient via tablet before or during the first clinic visit, with the option of a paper version or orally administered by a staff member as needed. Alternatively, the Distress Thermometer could be provided to the patient for completion in advance of the visit as part of their check-in or registration process for their appointment or during downtime in the waiting room.

After patients complete the screening, the non-physician oncology care provider leading the distress screening would upload and document the patients’ Distress Thermometer results within the Electronic Medical Record (EMR), notating both the patient’s Distress Thermometer score and Problem Checklist items that were marked. This staff member would relay results to the patient’s physician at the start of the visit. Following the initial visit, Distress Thermometer screenings would be conducted at every visit in which the patient is initiating a new course of treatment or beginning care with a new oncology physician (e.g., new radiation oncologists, surgical oncologists, and other medical oncologists). These financial distress re-screenings would also be led by a non-physician oncology care provider at the clinic according to the same procedures.

If financial distress is indicated, the non-physician oncology care provider would submit a referral to social work for financial toxicity assessment via the cancer center’s electronic system within 3–5 days of the visit. Social workers would subsequently schedule a full financial toxicity assessment using the 12-item Comprehensive Score for Financial Toxicity-Functional Assessment of Chronic Illness Therapy (COST-FACIT) version 2^42^ within 7–10 business days of receiving the request. Following this protocol, the referring staff member may ask physicians to triage specific patients for expedited assistance based on their professional judgment of potential severity of financial distress and the urgency of these issues in the context of the patient’s oncological treatment plan. When possible, follow-up phone calls or tele-visits with the primary oncology team may be advised in lieu of in-person visits to minimize patients’ travel expenses, co-pays, childcare expenses, disruption to work, etc.

Since 2015, the American College of Surgeons (ACoS) Commission on Cancer has mandated psychosocial distress screenings for accreditation of cancer centers.^43^ According to the National Comprehensive Cancer Network Guidelines Version 2.2023 Distress Management, psychological distress is defined as an emotionally unpleasant psychological (cognitive, behavioral, emotional), social, and/or spiritual experience that may interfere with a patient’s ability to effectively cope with cancer, its physical symptoms, and its treatment.^41^ The Distress Thermometer screens for the presence of psychosocial distress among cancer patients.^44^ The Distress Thermometer uses a 1-item Likert scale of distress from 0 (no distress) to 10 (extreme distress), with an accompanying list of causes.^45^ Recommended Distress Thermometer cutoff scores for detecting distress ranged from 2 to 7, with National Comprehensive Cancer Network guidelines indicating an optimal score of greater than or equal to 4, which varies by the differences in the components that comprise distress.^46^

Our investigator team used results from the current study and a comprehensive literature search to discuss and arrive at a recommendation that patients at the cancer center with Distress Thermometer screening results indicating a distress score >4 and >3 practical concerns on the Distress Thermometer problem list be referred by the health care provider who conducted the Distress Thermometer screening to the social work team fora full financial toxicity assessment using the COST-FACIT. Due to the urgency of some financial concerns, we suggest that health care providers make these referrals to the social work team within 3–5 business days of the Distress Thermometer screening.

### Financial Toxicity Assessment of Vulnerable Patients

The COST-FACIT is a standardized patient-reported outcome measure that has been validated to assess financial distress from cancer and its treatments.^3^ The COST-FACIT version 2 is a 12-item questionnaire asks patients to respond using a Likert scale.^3^ Since its introduction, the COST-FACIT has been shown to provide adequate reliability, validity, and internal consistency for identifying financial distress.^47^

Total scores of the COST-FACIT range from 0 to 48 and lower scores relate to higher patient-reported financial toxicity.^48^ While there is no established cutoff score with the COST-FACIT, studies have used median scores of 20 or 26 to indicate positive screenings for financial toxicity,^49^ while significant financial burden is often indicated by scores less than 22.^50^ In addition, Prasad et al’s study^51^ stratified gynecologic cancer patients as experiencing either mild (COST-FACIT score, 14–25), moderate (COST-FACIT score, 1–13), or severe (COST-FACIT score, 0) financial toxicity.

Our investigator team used results from the current study and a comprehensive literature search to discuss and arrive at procedures for financial toxicity assessment of patients at the cancer center. The COST-FACIT financial toxicity assessment would be led by a social worker who administers the assessment using the same format as the Distress Thermometer. Upon completion, the social worker would document the patients’ COST-FACIT results within the EMR and collaborate with the patient on initiating a relevant financial navigation strategy within 3–5 days of the COST-FACIT assessment. Next steps for financial strategies and a copy of the patients’ COST-FACIT assessment results would be made available to the patient via the electronic patient portal and made available to the referring oncology care provider via the staff electronic portal. The status on the execution of financial strategies would be updated by social workers on an ongoing basis via both the patient and provider electronic portals.

### Financial Navigation Pathway

Although medical financial hardship is not unique to cancer patients,^15,52,53^ the distinct nature of the cancer care continuum^48^ calls for interventions that optimize the oncological workflow among the interprofessional care team. Financial navigation of cancer patients is a feasible intervention that is utilized at comprehensive cancers centers across the country to reduce financial hardship.^54,55^ Service offerings that comprise a financial navigation program vary and may include financial toxicity screening, health insurance optimization, health literacy education, referral to social support services (e.g., housing, transportation, and other health related social needs), as well as financial budgeting and assistance programs.^56^ Given this wide range of offerings, an interprofessional approach and concerted collaboration between oncologists, primary care, social workers, and all other members of the interprofessional care team is necessary to arrive at meaningful strategies for the patient population at the cancer center.

**Figure 3.**
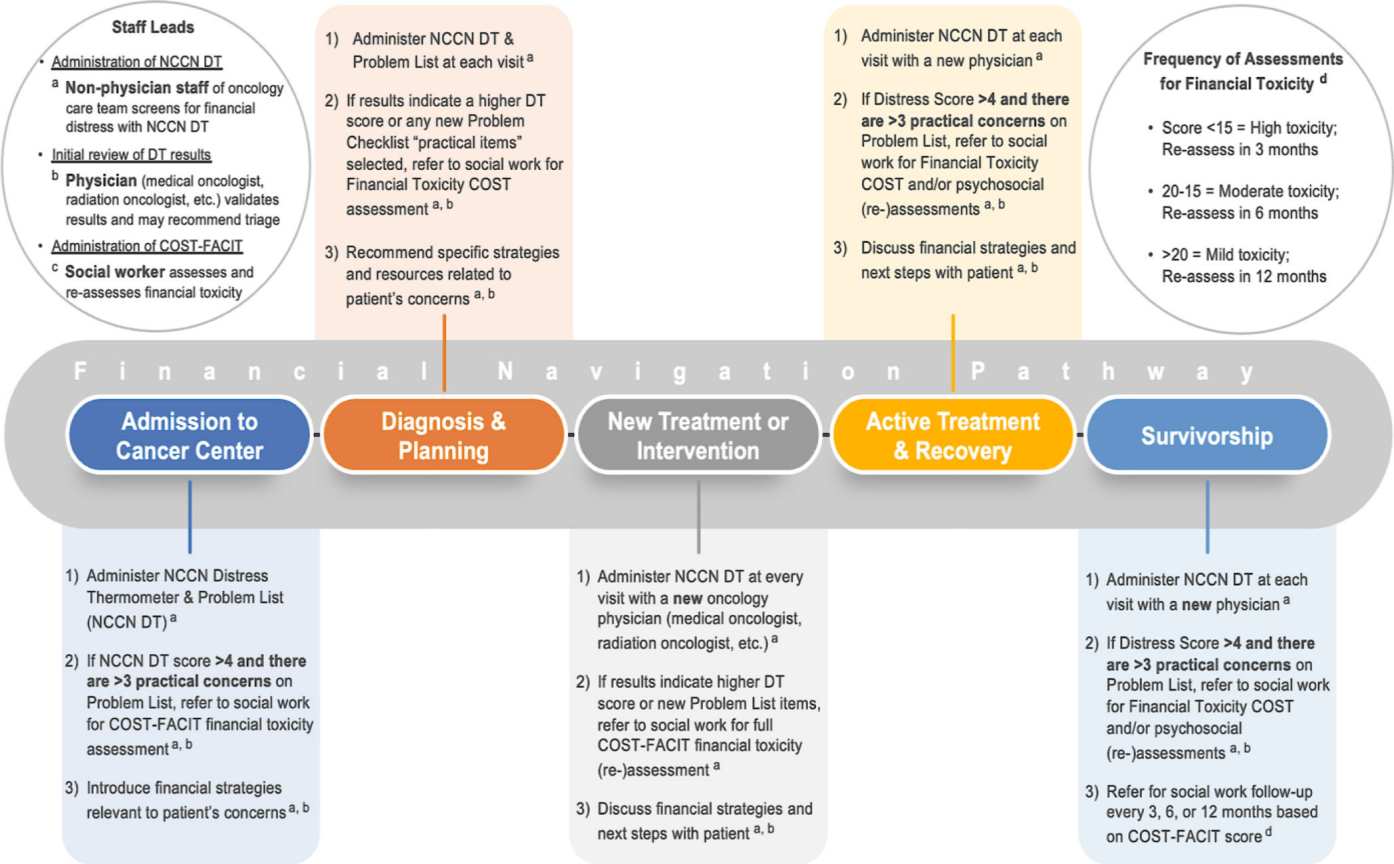
Pathway of financial navigation for health care providers to intervene on financial hardship

Figure 3 presents the financial navigation pathway for cancer patients at a National Cancer Institute designated comprehensive cancer center along the cancer care continuum that resulted from information on financial hardship experiences and when these hardships occurred. Across this financial navigation pathway, mapping the appropriate timing of financial strategies and ensuring consistent and timely communication among the interprofessional care team is essential.

*Anticipatory guidance* from cancer care professionals is warranted and strongly desired among patient participants who struggled with the financial consequences of cancer, and even more so among patients who reported pre-existing low income prior to their cancer diagnosis. Informed by these results, extant evidence, and clinical experience, qualified members of our research team in the current study recommend specific strategies for intervening upon each of these themes of financial hardship and the timing of these interventions along the cancer care continuum. Thus, the current study derived patient-centered, community-informed strategies from cancer patients and cancer care professionals to inform a sustainable financial navigation pathway for routine screening and intervention to reduce financial toxicity across the cancer care continuum at an NCI designated comprehensive cancer center (Figure 3).

### Practice Implications

Due to the strain of the global pandemic and post-pandemic consequences, cancer center staff have shared experiences of great shortfalls in staff capacity attributed to high turnover, burnout, and vacant positions. Given these significant challenges, the resulting financial navigation pathway may require practical adjustments to triage patients with the most severe needs and ensure that staff members at the cancer center have the capacity to sufficiently respond. Even with the potential need for more modest interim goals, the cancer center’s staff, patients, and their supporters will all benefit from a transparent process of financial navigation.

When adjusting to reflect reduced staff capacity, two areas for customization to ensure patient referrals of those with the greatest needs is in our recommendations for financial distress screening and financial toxicity assessment across the financial navigation pathway. Specifically, we suggest that Distress Thermometer screenings with scores of >4 be referred to social work for a financial toxicity assessment. This Distress Thermometer cutoff score for referrals may be adjusted higher to respond to the current staff capacity.

Similarly, we suggest that patients who score <20 on the COST-FACT receive more frequent re-assessment with patients scoring <15 to be re-assessed in 3 months while patients scoring 20–15 be re-assessed in 6 months. We also advise that all patients referred for financial toxicity assessment be re-assessed on a yearly basis at minimum. The recommended COST-FACIT cutoff scores and corresponding frequency of re-assessments may be adjusted according to patient needs and staff capacity of the clinic environment. Accordingly, patients experiencing the highest toxicity would be prioritized, and staff would be more likely to fully execute the suggested financial strategies at each re-assessment.

Communication and collaboration on staff roles and responsibility across the interdisciplinary care team is critical to the effectiveness of the financial navigation pathway. In the setting of a busy clinical practice, responsibilities that are workflow driven are more likely to be completed, and tasks that can be completed by qualified non-physician staff should be assigned accordingly. We recommend that screening for financial hardship be led by a non-physician member of the primary oncology care team (e.g., physician’s assistant, nurse practitioner, infusion/radiation nurse, etc.), who then makes referrals to social work for a full financial toxicity assessment. The specific staff assigned to this leadership role may be further narrowed or adjusted to reflect the staffing of the cancer center and optimize efficiency.

Equally important, the social work team must establish and monitor their capacity for responding to referrals. For example, we estimate that one full-time social worker at our cancer center has a weekly capacity to manage 6–9 patient referrals for financial toxicity assessment and subsequent financial navigation when considering the intensity of unmet needs among patients at our cancer center located in a dense urban setting. Staff workload can also be reduced by electronically administering re-assessments of either the Distress Thermometer screening or COST-FACIT financial toxicity assessment, since both are self-administered tools in which patients respond to questions independently.

### Limitations and Strengths

This study had limitations. Cancer patients who were interviewed in the study were treated at a National Cancer Institute designated comprehensive cancer center, which provided insights into the experiences of financial hardship for those who were able to access care. It does, however, exclude the perspectives of those who live within the cancer center’s catchment area, but are unable to access the cancer center and/or other medical care. It would have been helpful to include these patients not receiving care at the cancer center to examine potential barriers to accessing necessary medical care and their associations with financial toxicity.

Another limitation is that most patients (71 %) participating in this study were diagnosed with breast cancer and had curable disease that did not require chemotherapy. Also, over half of patient participants (54%) in our study held college or graduate degrees and reported incomes above this region’s median income of $54,124 between 2017–2021,^57^ The combination of lower disease burden, higher education attainment, and higher income levels suggest that we may have missed important aspect of financial toxicity from patients with severe needs.

Notwithstanding, these limitations are outweighed by our study’s strengths. This study makes a novel contribution to knowledge on patient-centered and community-informed approaches to developing a useful practice tool that can help cancer care providers to reduce the financial hardship of cancer experienced by patients in their care by leveraging existing services, resources, and interprofessional collaboration. The resulting financial navigation pathway establishes a systematic standard of care that creates equitable access to resources and strategies aimed at reducing the financial toxicity of cancer. By suggesting strategies that respond directly to the experiences and concerns expressed by cancer patients and cancer care professionals and optimizing the oncological workflow, we developed a tool that can be tested for effectiveness with a random sample of participants that could potentially address some of the limitations mentioned above.

## Conclusions

This study followed an innovative approach of centering the experiences of cancer patients and input from their care professionals to construct a financial navigation pathway tool that can be used in routine care at a National Cancer Institute designated comprehensive cancer center. Findings are a necessary first step toward developing a sustainable approach to reduce financial toxicity among cancer patients. It is the first in-depth investigation of patient-centered, community-informed financial navigation strategies at this NCI-designated comprehensive cancer center located in a dense urban area. Future research is needed to test the structures, processes, and outcomes of the financial navigation pathway tool; examine its impact on financial toxicity, and health-related outcomes; as well as better understand how much navigation is needed to bring about meaningful change.

## Figures and Tables

**Table 1.** Participant characteristics (n=50)^1^

Cancer patients (n=34)	Frequency	(%)
**Age at diagnosis**	43 (m)	12(SD)
26–39 years	14	41
40–65 years	18	53
65 years and over	1	3
**Age at time of study**	50 (m)	13(SD)
**Gender**		
Male	3	9
Female	29	85
Other	1	3
**Ethnicity**		
Not Hispanic	32	94
**Race**		
Asian	1	3
Black/African American	21	62
White/Caucasian	11	32
**Income**		
$24,999 and under	4	12
$25,000 through $49,999	6	18
$50,000 through $74,999	7	21
$75,000 and over	12	35
Don’t know	4	12
**Education level**		
High school diploma or equivalent	2	6
Some college	3	9
College degree	12	35
Graduate degree	16	47
**Cancer diagnosis**		
Breast cancer	24	71
Colon cancer	1	3
Prostate cancer	1	3
Esophageal cancer	1	3
Leukemia	1	3
Lung cancer	1	3
Lymphoma	1	3
Spinal Ependymoma	1	3
Unclassified blood cancer	1	3
Testis cancer	1	3
More than one cancer diagnosis	1	3
Cancer care professionals (n=16)	Frequency	(%)
**Age at time of study**	35 (m)	6 (SD)
**Gender**		
Male	3	19
Female	10	63
**Ethnicity**		
Latinx	2	13
Not Hispanic	11	69
**Race**		
Asian	2	13
Black/African American	1	6
White/Caucasian	9	56
Other	1	6
**Professional role**		
Social worker	3	19
Registered nurse	2	13
Nurse coordinator	1	6
Clinical nurse	2	13
Surgeon	3	19
Physician	3	19
Psychotherapist	1	6
Oncology researcher	1	6
**Years working in this role**	5 (m)	35 (SD)

1 Missing data due to no response

**Table 2.** Interview guides

*Questions for Cancer Patients*	
*Focus area*	*Questions for Patients*
Material issues	1. How did you experience financial challenges like making ends meet?
	2. How did your family or anyone in your household experience financial challenges like making ends meet?
Behavioral issues	3. What actions did you take to adjust to your new financial situation after a cancer diagnosis? For example, things that you started doing or stopped doing.
	4. What are actions that your family or anyone in your household took in order to adjust to your new financial situation after a cancer diagnosis?
Psychosocial issues	5. What are some of the emotional impacts that you’ve experienced, because of financial challenges after your cancer diagnosis?
	6. What are some of the emotional impacts that have come for your family or anyone in your household, because of financial challenges after your cancer diagnosis?
Participant financial support	7. How supportive were your family members to you financially?
Critical moments in survivorship when the financial hardship of cancer was experienced	8. Referring to the National Cancer Institute’s Cancer Care Continuum, can you describe particularly critical moments during the course of cancer detection, diagnosis, treatment, and survivorship, when you have experienced a lot of financial hardship?
	10. Can you describe a particularly critical time or times, when you were not going through active cancer treatments, when you experienced a lot of financial hardship?
	11. Can you describe particularly critical moments, when you were not actively on the cancer care continuum as pictured in this NCI diagram, when you’ve experienced a lot of financial hardship?
Financial actions, assistance, and resources that were helpful	12. What would you say are the most important financial actions that you or your family, or members of your household needed to take at the time when you were first diagnosed with cancer?
	13. Can you tell me about any assistance with financial challenges that you have utilized or found useful?
Advice for professionals to reduce financial hardship of cancer	14. If you were to give advice to staff at the Cancer Center what would be the top 3 pieces of advice that you would share about handling the financial hardships of their cancer patients?
Advice for other patients to reduce financial hardship of cancer	15. Similarly, if you were to advise another patient who was just diagnosed with cancer, what would be the top 3 pieces of advice that you would give about handling their financial hardships?
*Questions for Cancer Care Professionals*	
*Focus area*	*Questions for Cancer care Professionals*
Material issues	1. How did your patients experience financial challenges like making ends
	2. How did your patient’s families or anyone in their household experience financial challenges like making ends meet?
Behavioral issues	3. What are actions that patients have taken to adjust to their new financial situation after a cancer diagnosis? For example, things that they started doing or stopped doing.
	4. What are actions that your patients’ families or anyone in their household took to adjust to their new financial situation after a cancer diagnosis?
Psychosocial issues	5. What are some of the emotional impacts that you have observed in your patients because of the financial challenges following a cancer diagnosis?
	6. What are some of the emotional impacts that you have observed in your patients’ family or anyone in their household because of the financial challenges following a cancer diagnosis?
Critical moments in survivorship when the financial hardship of cancer was experienced	7. Referring to the National Cancer Institute’s Cancer Care Continuum, can you describe particularly critical moments during the course of cancer detection, diagnosis, treatment, and survivorship, when your patients have experienced a lot of financial hardship?
	8. Can you describe particularly critical moments, when your patients were not actively on the cancer care continuum as pictured in this NCI diagram, when they experienced a lot of financial hardship?
Helpful financial actions, assistance, and resources	9. What would you say are the most important financial actions that your patients or their families, or members of their households needed to take at the time when they were first diagnosed with cancer?
	10. Can you tell me about any financial assistance that you have utilized or found useful for your patients and/or their household members?
Advice for other professionals to reduce financial hardship of cancer	11. If you were to give advice to your colleagues at the Cancer Center what would be the top 3 pieces of advice that you would share about handling the financial hardships of their cancer patients?
Advice for patients to reduce financial hardship of cancer	12. Similarly, if you were to advise patients who were just diagnosed with cancer, what would be the top 3 pieces of advice that you would give about handling their financial hardships?

**Table 3.** Representative quotations of the financial hardship of cancer

**Material Domain—i.e., Financial challenges in making ends meet**
*Patients*
“Out of like my AmeriCorps insurance, I got it as long as I could. And then I for the rest of that year, I paid health insurance out of my pocket. Because it was better insurance than what I could get, like going on whatever disability like Medicare, whatever. So, I did that. And that completely depleted my savings.”
“It was challenging to make ends meet. And that does include not going to doctor’s visits … nobody can live off one bra.”
“Once I was post-treatment, my job … they fired me because of my cancer … that’s where the hardship actually kind of began.”
“I did live with my parents during treatment because I needed care…they were already paying for my food; they were already paying for like transportation costs. I mean, they didn’t ask me for anything, they paid for the gas, they paid for, they drove, and they picked up medication.”
*Cancer Care Professionals*
“We see a lot of patients whose parents might either stop working completely or take you know, an extended unpaid leave from work. And we see a lot of associated hardships with that financially. You know, trouble making ends meet to pay their rent, trouble, you know, paying for utilities.”
“I’ve had single people tell me issues with employment. And then there’s kind of some more unspoken issues when you can tell patients are struggling because they’re missing appointments, they can’t find rides to and from the Cancer Center.”
“A lot of resentment between spouses. You know, there’s already like, some problems there with transitioning from being like lovers’ spouses, almost into like a parental role in a way because they’re having to care for their loved one.”
**Behavioral Domain—i.e., Efforts to adjust to new financial situation**
*Patients*
“I stopped doing a lot of impulse buying, you know, luxury items, clothing, and stuff like that, I just thought that it could be a lot of things, but one, I didn’t really have the energy to go out, like I used to.”
“I was going through treatment. So of course, my mom was like, you know, buying me like expensive like organic food.”
“I needed my mom’s support while 1 was recovering from surgery and stuff. So, there were times that she was home with me without receiving pay … picking up medication and stuff during that time.”
*Cancer Care Professionals*
“I noticed just like a lot of families not being able to afford to, you know, go to summer camp during the summertime or yeah, definitely family vacations, those changes. And even like food, you know, maybe your food changes to where you’re not eating, you know, the same foods that you did before because you can’t afford them. So, you’re staying home more, you’re buying cheaper options, more processed foods, those type of changes.”
“So, I think one of the toughest tradeoffs that I’ve actually heard from my own work is the fact that some cancer patients may actually decide not to get treated, or not to continue their treatment in order to be able to pay for these needs.”
“Kids won’t be able to, you know, purchase the shoes that they need, or the equipment that they need for their sports or, you know, if it’s an instrument, they’re not able to play their instrument anymore, because it costs a certain amount.”
“But for a lot of them, they often have to take off from work as well to either pick up a relative from a session or take a relative to a session, or even if it’s not a session related to cancer treatment, they still have other appointments as well.”
**Psychosocial Domain—i.e., Stresses that come from not making ends meet**
*Patients*
“It’s a different kind of helplessness. A big thing that cancer does is it takes away a lot of your control.”
“The main emotional impact was I was always wondering ‘At what point would I be too sick to work?’ But the emotional impact again becomes how does it impact my family? … So that right there that right now has been weighing on my mind, like how are we going to manage to pay our bills and doing activities and things like that, without the income that we’ve always had?”
“And then they put some additional stress on my husband with making sure that everything that we need is taken care of, in addition to my health, so that’s a little stressful trying to, you know, navigate how it affects everybody as a whole.”
“So, they’re hyper aware of when we are spending money and how much we are spending. And that’s not something I ever wanted them to see or experience because I had to do that when I was little not related to cancer, but like I know what that feels like as a kid.”
*Cancer Care Professionals*
“It’s just like a point of stress and frustration. A lot of people get angry. I’ve seen even the nicest patients when their treatments interfere with their work schedules, they just get angry.”
“So, I mean, it’s the natural thing is just like, sorrow and, like distress, just like with the stress of the bills and everything like that, in addition to having like this deadly diagnosis that you also have to deal with.”
“And I think it’s also hard for the family too because you already have caregiver burden on top of that, and burnout.”
“They feel distressed about asking for help from family members … They don’t want that type of impact on their relationships or just kind of feeling a sense of pride and not wanting to ask.”

**Table 4.** Common themes on cancer patients for each domain of financial hardship^1^

Material domain—i.e., Financial challenges in making ends meet		
*Key themes on cancer patients*		*Strategies from cancer care professionals*
Theme 1	Insufficient, lack of, or loss of health insurance and benefits as low-wage earner	Review eligibility for insurance coverage and pharmacy benefit programs; Refer to financial advisor for audit, navigation, etc.
Theme 2	Health insurance issues with mounting medical bills and household bills	Establish relationships and a routine communications plan wit all care managers involved with patient; Refer to financial advisor for audit, navigation, etc.
Theme 3	Employment problems including job loss, job lock, and inability to work due to cancer	Provide Family Medical Leave Act (FMLA) forms to patients and caregivers; Refer to financial advisor for audit, navigation, etc.
Theme 4	Higher food costs for tolerable or healthier choices	Provide information on appropriate nutrition, resources to access recommended foods, and tips on food cost-savings
Theme 5	Unable to pay bills for utilities and other basic needs that persisted	Facilitate access to direct financial support programs as needed (e.g., Utilities assistance programs; philanthropic sources for mini-grants or funds)
Theme 6	Divested from financial savings	Recommend participation in financial literacy group
Theme 7	New or increased non-medical expenses to manage side effects	Recommend affordable options; Review eligibility and utility of existing benefits to meet new financial needs (e.g., flexible spending accounts; Facilitate access to relevant financial support programs)
Theme 8	Loss of personal property by selling or having it repossessed	Provide hardship letter as needed (e.g., to mortgage lender; Refer to social work for financial counseling)
Theme 9	Received financial support from personal networks	Refer to social work for psychosocial (re-)assessment of available informal support and recommendations on recruiting help from social networks
Theme 10	Received financial support from program or organization	Provide a financial resource guide
Behavioral domain—i.e., Efforts to adjust to new financial situation		
*Key themes on cancer patients*		*Strategies from cancer care professionals*
Theme 1	Reduced or stopped personal discretionary expenses	Follow direction of patient or caregiver and provide guidance, referrals, and support as needed
Theme 2	Took on additional work to create new income	Follow direction of patient or caregiver and provide guidance, referrals, and support as needed
Theme 3	Changed living situation	Follow direction of patient or caregiver and provide guidance, referrals, and support as needed
Theme 4	Changed personal financial priorities; Prioritized affording basic needs over medical care	Follow direction of patient or caregiver and provide guidance, referrals, and support as needed; Initiate discussion of patient’s ability to afford basic needs
Theme 5	Negotiated payment plan for medical expenses	Follow direction of patient or caregiver and provide guidance, referrals, and support as needed
Theme 6	Nonadherence to follow-up appointments and care	Inquire about and identify other priorities/barriers/misinformation that may lead to not attending follow-up appointments
Theme 7	Nonadherence to prescribed medications to spread out doses over longer period	Initiate early identification of patient’s need for financial assistance with drug coverage
Psychosocial domain—i.e., Stresses that come from not making ends meet		
*Key themes on cancer patients*		*Strategies from cancer care professionals*
Theme 1	Exacerbated the emotional stress of having cancer; Experienced anger and frustration with treatment	Refer to individual or group psychotherapy; Refer to social work for follow-up; Refer to primary care physician for follow-up
Theme 2	Increased financial worries, anxiety, and fears due to financial situation	Refer to individual or group psychotherapy; Refer to social work for follow-up; Refer to primary care physician for follow-up
Theme 3	Strained personal relationships	Refer to individual or group psychotherapy; Refer to social work for follow-up; Refer to primary care physician for follow-up
Theme 4	Stress about being a financial burden and sorrow due to financial hardship	Refer to individual or group psychotherapy; Refer to social work for follow-up; Refer to primary care physician for follow-up
Theme 5	Stress about employment implications, work interference with treatments, and premature return to work	Refer to individual or group psychotherapy; Refer to social work for follow-up; Refer to primary care physician for follow-up
Theme 6	Feelings of guilt or shame about having cancer	Refer to individual or group psychotherapy; Refer to social work for follow-up; Refer to primary care physician for follow-up
Theme 7	Suicidal thoughts	Refer to individual or group psychotherapy; Refer to social work for follow-up; Refer to primary care physician for follow-up

1 Cheung, C. K., Nishimoto, P. W., Katerere-Virima, T., Helbling, L. E., Thomas, B. N., & Tucker-Seeley, R. (2022). Capturing the financial hardship of cancer in military adolescent and young adult patients: A conceptual framework. *Journal of psychosocial oncology, 40*(4), 473–490. https://doi.org/10.1080/07347332.2021.1937771: Tucker-Seeley, R. D., & Thorpe, R. J. (2019). Material-Psychosocial-Behavioral Aspects of Financial Hardship: A Conceptual Model for Cancer Prevention. The Gerontologist, 59(Suppl 1), S88-S93. https://doi.org/10.1093/geront/gnz033

**Table 5.** Common themes on family/household members of cancer patients for each domain of financial hardship^1^

Material domain—i.e., Financial challenges in making ends meet		
*Key themes on family/household members*		*Recommended strategies from cancer care professionals*
Theme 1	Family members asked to contribute practical support and felt pressured to help	Refer caregiver to local support groups, psychotherapy, classes, and other resources; Offer family members/caregivers opportunities to participate in pro bono financial services (e.g., financial counseling and advising)
Theme 2	Family members asked to make financial contributions and felt burdened	Refer to social work for financial audit and counseling on appropriate options and strategies; Offer family members/caregivers opportunities to participate in pro bono financial services (e.g., financial counseling and advising)
Theme 3	Increasing needs from financial dependents	Refer to social work for financial audit and counseling on appropriate options and strategies
Theme 4	Job loss experienced by family or household members	Refer to social work for financial audit and counseling on appropriate options and strategies; Assist with unemployment application
Theme 5	Family members traveled great distances to provide support	Refer to social work for follow-up on available temporary housing programs and assistance for patient and their families traveling to treatment; Modify treatment schedule to optimize Cancer Center visits and minimize travel expenses
Theme 6	Family members divested from savings to contribute financially	Refer to social work for financial audit and counseling on appropriate options and strategies
Theme 7	Little to no support from family or household	Refer patient to social work for comprehensive psychosocial assessment and connection to services and support as needed
Behavioral domain—i.e., Efforts to adjust to new financial situation		
*Key themes on family/household members*		*Recommended strategies from cancer care professionals*
Theme 1	Took on new financial responsibilities and additional work due to financial struggle	Follow direction of patient or caregiver and provide guidance, referrals, and support as needed; Offer family/household members opportunity to participate in introductory financial counseling and resources for pro bono financial advising
Theme 2	Provided practical support to cancer patient (e.g., cooking, cleaning, transportation for daily activities among family members, etc.)	Follow direction of patient or caregiver and provide guidance, referrals, and support as needed; Suggest sustainable ways practical support can be provided following the lead and/or preferences of patient and their caregiver(s)
Theme 3	Provided emotional support to cancer patient	Follow direction of patient or caregiver and provide guidance, referrals, and support as needed
Theme 4	Provided medical assistance to cancer patient (e.g., transportation to medical appointments, organizing medications)	Follow direction of patient or caregiver and provide guidance, referrals, and support as needed; Suggest sustainable ways practical support can be provided following the lead and/or preferences of patient and their caregiver(s)
Theme 5	Discontinued activities that nurture family ties	Offer financial advising for budget development or revision with allocation of funds for discretionary expenses
Theme 6	Helped patient to pay bills	Offer family members/caregivers opportunities to participate in pro bono financial services (e.g., financial counseling and advising)
Theme 7	Contacted providers to advocate for patient	Suggest ways advocacy can be provided following patient/caregiver initiation and preferences
Psychosocial domain—i.e., Stresses that come from not making ends meet		
*Key themes on family/household members*		*Recommended strategies from cancer care professionals*
Theme 1	Emotional conflicts among family and/or household members	Refer to individual or group psychotherapy; Refer to social work for follow-up; Refer to primary care physician for follow-up
Theme 2	Family member(s) provided emotional support to patient	Refer to individual or group psychotherapy; Refer to social work for follow-up; Refer to primary care physician for follow-up
Theme 3	Children became worried about finances	Refer to individual or group psychotherapy; Refer to social work for follow-up; Refer to primary care physician for follow-up
Theme 4	Strain in family relationships due to role changes and caregiving	Refer to individual or group psychotherapy; Refer to social work for follow-up; Refer to primary care physician for follow-up
Theme 5	Distressed about asking others for help	Refer to individual or group psychotherapy; Refer to social work for follow-up; Refer to primary care physician for follow-up
Theme 6	Personal mental health struggles with anxiety and depression	Refer to individual or group psychotherapy; Refer to social work for follow-up; Refer to primary care physician for follow-up
Theme 7	Burnout from providing practical support	Refer to individual or group psychotherapy; Refer to social work for follow-up; Refer to primary care physician for follow-up

1 Cheung, C. K., Nishimoto, P. W., Katerere-Virima, T., Helbling, L. E., Thomas, B. N., & Tucker-Seeley, R. (2022). Capturing the financial hardship of cancer in military adolescent and young adult patients: A conceptual framework. *Journal of psychosocial oncology, 40*(4), 473–490. https://doi.org/10.1080/07347332.2021.1937771: Tucker-Seeley, R. D., & Thorpe, R. J. (2019). Material-Psychosocial-Behavioral Aspects of Financial Hardship: A Conceptual Model for Cancer Prevention. The Gerontologist, 59(Suppl 1), S88-S93. https://doi.org/10.1093/geront/gnz033

**Table 6.** Emergent themes and subthemes for intervening on financial hardship across the cancer care continuum^1^

Theme 1	Critical moments in survivorship when the financial hardship of cancer was experienced
Subtheme 1	Personal financial hardship of being low-income co-occurred
Subtheme 2	Side effects surfaced
Subtheme 3	Less informal support was present
Subtheme 4	Need for continuing medical care, follow-up, and other costs after active treatment
Subtheme 5	Bills accumulated during treatment are due
Subtheme 6	Returning to work or navigating new career
Subtheme 7	Paying for ongoing medications
Subtheme 8	Cancer recurrence
Subtheme 9	Increased supportive care needs
Subtheme 10	Transportation and parking costs during treatment
Subtheme 11	Housing costs during and after treatment
Subtheme 12	Palliative care decision-making
Subtheme 13	Detection and diagnosis due to pre-existing financial hardship
Theme 2	Financial assistance and resources that were helpful
Subtheme 1	Direct financial support from family
Subtheme 2	Family or friends helped to access financial resources
Subtheme 3	Family member’s health insurance
Subtheme 4	Professional help finding financial assistance
Subtheme 5	Practical resources for cancer survivorship
Subtheme 6	Patient education on financial assistance programs
Subtheme 7	Financial assistance through grant programs
Subtheme 8	Financial support for specific medical issues
Subtheme 9	Disability insurance
Subtheme 10	Practical support for specific medical issues
Subtheme 11	Cancer-specific programs and services
Subtheme 12	Financial grants to support psychosocial needs
Theme 3	Advice to staff at the Cancer Center for reducing the financial hardship of cancer
Subtheme 1	Increase awareness of patients’ social and financial circumstances
Subtheme 2	Increase knowledge of patient needs before treatment
Subtheme 3	Provide financial information before treatment begins
Subtheme 4	Provide emotional support
Subtheme 5	Provide equal services regardless of patients’ race
Subtheme 6	Offer peer support
Subtheme 7	Initiate difficult conversations and ask patients often about financial hardship
Subtheme 8	Facilitate access to support groups
Subtheme 9	Provide information on grant programs and financial assistance
Subtheme 10	Routinely assess patients’ financial situation
Subtheme 11	Advocate for financial resources on behalf patients and at your institution
Subtheme 12	Listen and respond to patients’ concerns and needs
Subtheme 13	Create welcoming environment for financial hardship conversations
Subtheme 14	Know financial resources and how to access and utilize
Theme 4	Advice to other patients for reducing the financial hardship of cancer
Subtheme 1	Ask for practical and financial help
Subtheme 2	Create robust professional and personal networks of support
Subtheme 3	Contact insurance providers to understand your coverage and resources
Subtheme 4	Advocate strongly for yourself and ask for financial help from cancer care team
Subtheme 5	Ask questions about finances
Subtheme 6	Apply for financial support and document these efforts
Subtheme 7	Meet with social workers and other supportive care staff
Subtheme 8	Prioritize focusing on treatment and maintaining a healthy lifestyle
Subtheme 9	Identify disease-specific resources
Subtheme 10	Take account of personal support system
Subtheme 11	Reach out to healthcare providers for financial assistance

1 National Institutes of Health, (n.d.). Cancer control continuum. Cancer Control Continuum | Division of Cancer Control and Population Sciences (DCCPS). Retrieved October 13, 2022, from https://cancercontrol.cancer.gov/about-dccps/about-cc/cancer-control-continuum

## References

[1] Pangestu S, Rencz F. Comprehensive score for financial toxicity and health-related quality of life in patients with cancer and survivors: A systematic review and meta-analysis. Value Health J Int Soc Pharmacoeconomics Outcomes Res. 2023;26(2):300–316. doi: 10.1016/j.jva1.2022.07.017 36064514

[2] PDQ Adult Treatment Editorial Board. Financial Toxicity and Cancer Treatment (PDQ^®^): Health Professional Version. In PDQ Cancer Information Summaries. National Cancer Institute. Published 2022. Accessed August 31, 2023. https://www.cancer.gov/about-cancer/manaqinq-care/track-care-costs/financial-toxicity-pdq

[3] de Souza JA, Yap BJ, Wroblewski K, Measuring financial toxicity as a clinically relevant patient-reported outcome: The validation of the Comprehensive Score for doi:10.1002/cncr.30369 PMC5298039 27716900

[4] Ehsan AN, Wu CA, Minasian A, Financial toxicity among patients with breast cancer worldwide: A systematic review and meta-analysis. JAMA Netw Open. 2023; 6(2):e2255388. doi: 10.1001/jamanetworkopen.2022.55388 PMC9909501 36753274

[5] Harris JP, Ku E, Harada G, Severity of financial toxicity for patients receiving palliative radiation therapy. Am J Hosp Palliat Care. Published online July 5, 2023: 10499091231187999. doi:10.1177/10499091231187999 PMC10772523 37406195

[6] Surachman A, Tucker-Seeley R. & Almeida DM (2023). The association between material-psychological-behavioral framework of financial hardship and markers of inflammation: A cross-sectional study of the Midlife in the United States (MIDUS) Refresher cohort. BMC Public Health, 23(1), 1845. 10.1186/s12889-023-16745-x 37735377 PMC10514981

[7] Kl Alcaraz, Wiedt TL Daniels EC, Yabroff KR, Guerra CE, Wender RC. Understanding and addressing social determinants to advance cancer health equity in the United States: A blueprint for practice, research, and policy. CA Cancer J Clin. 2020;70(1):31–46. doi: 10.3322/caac.21586 31661164

[8] Biddell CB, Waters AR, Angove RSM, Facing financial barriers to healthcare: patient-informed adaptation of a conceptual framework for adults with a history of cancer. Front Psychol. 2023;14. Accessed August 31, 2023. https://www.frontiersin.org/articles/10.3389/fpsyg.2023.1178517 10.3389/fpsyg.2023.1178517 PMC10225523 37255517

[9] Benedict C, Fisher S, Schapira L, Greater financial toxicity relates to greater distress and worse quality of life among breast and gynecologic cancer survivors. Psychooncology. 2022;31 (1 ):9–20. doi:10.1002/pon.5763 34224603 PMC9809212

[10] Ml Liang, Harrison R Aviki EM, Esselen KM, Nitecki R, Meyer L. Financial toxicity: A practical review for gynecologic oncology teams to understand and address patient-level financial burdens. Gynecol Oncol. 2023;170:317–327. doi:10.1016/j.ygyno.2023.01.035 36758422

[11] Haier J, Schaefers J. Economic perspective of Cancer Care and Its Consequences for Vulnerable Groups. Cancers. 2022; 14(13):3158. doi: 10.3390/cancers14133158 35804928 PMC9265013

[12] 9. Hong YR, Salloum RG, Yadav S, Smith G, Mainous AG. Patient-provider discussion about cancer treatment costs and out-of-pocket spending: Implications for shared decision making in cancer care. Value Health. doi:10.1016/j.jval.2020.08.002 33248514

[13] Jagsi R, Momoh AO, Qi J, Impact of radiotherapy on complications and patient-reported outcomes after breast reconstruction. J Natl Cancer Inst. 2018;110(2):157–165. doi:10.1093/jnci/djx148 28954300 PMC6059091

[14] Kronfli D, Savia B, Lievers A, Identifying psychosocial needs of patients with cancer undergoing curative radiation therapy in an inner-city academic center to address racial disparities. Int J Radiat Oncol Biol Phys. 2022;114(2): 185–194. doi: 10.1016/j.ijrobp.2022.04.003 35490990

[15] Khera N, Holland JC, Griffin JM. Setting the stage for universal financial distress screening in routine cancer care. Cancer. 2017;123(21 ):4092–4096. doi:10.1002/cncr.30940 28817185

[16] Delgado-Guay M, Ferrer J, Rieber AG, Financial distress and its associations with physical and emotional symptoms and quality of life among advanced cancer patients. The Oncologist. 2015;20(9):1092–1098. doi: 10.1634/theoncologist.2015-0026 26205738 PMC4571810

[17] Lee EM, Jiménez-Fonseca P, Hernández R, The role of financial difficulties as a mediator between physical symptoms and depression in advanced cancer patients. Curr Oncol. 2023;30(6):5719–5726. doi: 10.3390/curroncol30060429 37366912 PMC10297664

[18] Bradley CJ, Yabroff KR, Zafar SY, Shih YCT. Time to add screening for financial hardship as a quality measure? CA Cancer J Clin. 2021; 71 (2):100–106. doi:10.3322/caac.21653 33226648 PMC9116031

[19] Jacobsen PB, Ransom S. Implementation of NCCN distress management guidelines by member institutions. J Natl Compr Cancer Netw JNCCN. 2007;5(1):99–103. doi:10.6004/jnccn.2007.0010 17239329

[20] Dreismann L, Schoknecht K, Vogel A, Zimmermann T. Should I call psycho-oncology? Training nurses on psycho-oncological screening reduces uncertainties. J Cancer Res Clin Oncol. 2023;149(12):10585–10592. doi:10.1007/s00432-023-04936-3 37291403 PMC10423155

[21] Mitchell AJ, Hussain N, Grainger L, Symonds P. Identification of patient-reported distress by clinical nurse specialists in routine oncology practice: a multicentre UK study. Psychooncology. 2011;20(10):1076–1083 doi:10.1002/pon.1815 20687195

[22] Werner A, Stenner C, Schüz J. Patient versus clinician symptom reporting: how accurate is the detection of distress in the oncologic after-care? Psychooncology. 2012;21 (8):818–826. doi:10.1002/pon.1975 21544897

[23] Donovan KA, Grassi L, Deshields TL, Corbett C, Riba MB. Advancing the science of distress screening and management in cancer care. Epidemiol Psychiatr Sci. 2020;29:e85. doi:10.1017/S2045796019000799 31915097 PMC7214715

[24] McLouth LE, Nightingale CL, Dressier EV, Current practices for screening and addressing financial hardship within the NCI community oncology research program. Cancer Epidemiol Biomark Prev Publ Am Assoc Cancer Res Cosponsored Am Soc Prev Oncol. 2021; 30(4):669–675. doi:10.1158/1055-9965.EPI-20-1157 PMC8026561 33355237

[25] Smith SK, Nicolla J, Zafar SY. Bridging the gap between financial distress and available resources for patients with cancer: a qualitative study. J Oncol Pract. 2014;10(5):e368–372 doi:10.1200/jop.2013.001342 24865219 PMC5706140

[26] Corrigan KL, Fu S, Chen YS, Financial toxicity impact on younger versus older adults with cancer in the setting of care delivery Cancer. 2022;128(13):2455–2462 doi:10.1002/cncr.34220 35417565 PMC9177670

[27] Hastert TA, Young GS, Pennell ML, Financial burden among older, long-term cancer survivors: Results from the LILAC Study. Cancer Med. 2018;7(9):4261–4272. doi:10.1002/cam4.1671 30019387 PMC6143934

[28] Imber BS, Varghese M, Ehdaie B, Gorovets D. Financial toxicity associated with treatment of localized prostate cancer. Nat Rev Urol. 2020;17(1):28–40. doi: 10.1038/s41585-019-0258-3 31792431 PMC8010900

[29] Kent EE, Forsythe LP, Yabroff KR, Are survivors who report cancer-related financial problems more likely to forgo or 119(20):3710–3717. doi:10.1002/cncr.28262 PMC4552354 23907958

[30] Cheung CK, Nishimoto PW, Katerere-Virima T, Helbling LE, Thomas BN, Tucker-Seeley R. Capturing the financial hardship of cancer in military adolescent and young adult patients: A conceptual framework. J psychosoc Oncol. 2022;40(4):473–490. Doi:10.1080/07347332.2021.1937771 34152263

[31] Tucker-Seeley RD, Thorpe RJ Jr. Material-psychosocial-behavioral aspects of financial hardship: A conceptual model for Cancer prevention. The Gerontologist. 2019;59(supplement_1):S88–S93 doi:10.1093/geront/gnz033 31100144 PMC6524757

[32] Hp Freeman. The origin, evolution, and principles of patient navigation. Cancer Epidemiol Biomarkers prev.2012;21(10):1614–1617. Doi:10.1158/1055-9965.EPI-12-0982 23045534

[33] Donabedian A. Evaluating the quality of medical care. Milkbank Mem Fund Q. 1966;44(3):166–206. doi:10.2307/3348969 5338568

[34] Donabedian A. The quality of care: How Can it be assesses? JAMA. 1988;260(12): 1743–1748 doi:10.1001/jama.1988.03410120089033 3045356

[35] Donabedian A. Evaluating the quality of medical care. MilbankQ. 2005;83(4):691–729. Doi:10.1111/j.1468-0009.2005.00397.x PMC2690293 16279964

[36] Peterson K, Anderson J, Bourne D, Health care coordination theoretical frameworks: A systematic scoping review to increase their understanding and use in practice. J Gen Intern Med. 2019;34(1):90–98. doi:10.1007/s11606-019-04966-z PMC6542910 31098976

[37] Tossaint-Schoenmakers R, Versluis A, Chavannes N, Talboom-Kamp E, Kasteleyn M. The challenge of integrating ehealth into health care: Systematic literature review of the Donabedian model of structure, process, and Outcome. J Med Internet Res. 2021;23(5):e27180. doi:10.2196/27180 PMC8145079 33970123

[38] Jw Creswell. Qualitative Inquiry and Research Design: Choosing among Five Apporaches, 2nd Ed. Sage Publications, Inc; 2007:xvii, 395

[39] Gale NK, Heath G, Cameron E, Rashid S, Redwood S. Using the framework method for the analysis of qualitative data in multi-disciplinary health research. BMC Med Res Methodol. 2013;13:117. Doi:10.1186/1471-2288-13-117 24047204 PMC3848812

[40] National Cancer Institute. Cancer Control Continuum. National Cancer Institute Division of Cancer Control and Population Sciences. Accessed August 31, 2023. https://cancercontrol.cancer.gov/about-dccps/about-cc/cancer-control-continuum

[41] National Comprehensive Cancer Network. NCCN Guidelines Version 2.2023 Distress Management. Accessed August 31, https://www.nccn.org/docs/default-source/patient-resources/nccn_distress_thermometer.pdf

[42] de Souza JA, Yap BJ, Hlubocky FJ, The development of a financial toxicity patient-reported outcome in cancer: The COST measure. Cancer. 2014;120(20):3245–3253. doi: 10.1002/cncr.28814 24954526

[43] Pirl WF, Fann JR, Greer JA, Recommendations for the implementation of distress screening programs in cancer centers: Report from the American Psychosocial Oncology Society (APOS), Association of Oncology Social Work (AOSW), and Oncology Nursing Society (ONS) joint task force. Cancer. 2014;120(19):2946–2954. doi:10.1002/cncr.28750 24798107

[44] Pizarro DMP, Francia MBI, Ordinario MVC. Diagnostic accuracy of the NCCN distress thermometer for the assessment of psychosocial distress among Filipino patients with cancer. Acta Med Philipp. 2022;56(12). doi:10.47895/amp.viO.3463

[45] Alanazi AK, Lynch-Kelly D, Weaver M, Lyon DE. A scoping review of psychological distress instruments in women with early-stage breast cancer during chemotherapy. Cancer Rep. 2023;6(6):e1833. doi:10.1002/cnr2.1833 PMC10242653 37170774

[46] McElroy JA, Waindim F, Weston K, Wilson G. A systematic review of the translation and validation methods used for the national comprehensive cancer network distress thermometer in non-English speaking countries. Psychooncology. 2022;31 (8):1267–1274. doi: 10.1002/pon.5989 35757974

[47] Pavela G, Fifolt M, Tison S, Allison M, Burton B, Ford E. Re-validation of the Comprehensive Score for financial Toxicity (COST): Assessing the scale’s utility in chronic disease populations. Health Serv Insights. doi:10.1177/11786329211057352 PMC8669122 34916802

[48] D’Rummo KA, Nganga D, Chollet-Hinton L, Shen X. Comparison of two validated instruments to measure financial hardship in cancer survivors: comprehensive score for financial toxicity (COST) versus personal financial wellness (PFW) scale. Support Care doi:10.1007/s00520-022-07455-y 36513902

[49] Dee EC, Chino F. Financial hardship in cancer care—The need to define and intervene on actionable metrics. JAMA Netw Open. 2022;5(7):e2223149. doi: 10.1001/jamanetworkopen.2022.23149 35877128

[50] Ver Hoeve ES, Ali-Akbarian L, Price SN, Lothfi NM, Hamann HA. Patient-reported financial toxicity, quality of life, and health behaviors in insured US cancer survivors. Support Care Cancer Off J Multinatl Assoc Support Care Cancer. 2021; 29(1 ):349–358. doi:10.1007/s00520-020-05468-z PMC9208736 32361832

[51] Prasad RN, Patel TT, Keith SW, Eldredge-Hindy H, Fisher SA, Palmer JD. Development of a financial toxicity screening tool for radiation oncology: A secondary analysis of a pilot prospective patient-reported outcomes study. Adv Radiat Oncol. 2021; 6(6):100782. 10.1016/j.adro.2021.100782 PMC8503853 34660939

[52] Patel MR, Zhang G, Heisler M, Measurement and validation of the comprehensive score for financial toxicity (COST) in a population with diabetes. Diabetes Care. 2022;45(11):2535–2543. doi:10.2337/dc22-0494 36048837 PMC9679256

[53] Wang SY, Valero-Elizondo J, AM H, Out-of-pocket annual health expenditures and financial toxicity from healthcare costs in patients with heart failure in the United States. J Am Heart Assoc. 2021; 10(14):e022164. doi:10.1161/JAHA.121.022164 PMC8483501 33998273

[54] Shankaran V, Leahy T, Steelquist J, Pilot feasibility study of an oncology financial Navigation program. J Oncol Pract. 2018; 14(2):e122–e129. doi:10.1200/J0p.2017.024927 29272200

[55] Wheeler S, Rodriguez-O’Donnell J, Rogers C, Reducing cancer-related financial toxicity through financial navigation: Results from a pilot intervention. Cancer Epidemiol Biomakers Prev. 2020;29:694.2–694 doi:10.1158/1055-9965.EPI-20-0067

[56] Khan HM, Ramsey S, Shankaran V. Financial toxicity in cancer care: Implications for clinical care and potential practice solutions. J Clin Oncol. 2023:41(16):3051–3058 doi:10.1200/JC0.22.01799 37071839

[57] United States Census Bureau. QuickFacts Baltimore city, Maryland. Published 2021. Accessed August 9, 2023. https://www.census.gov/quickfacts/fact/table/baltimorecitymaryland/INC110221

